# Patients with limitation or withdrawal of life supporting care admitted in a medico-surgical intermediate care unit: Prevalence, description and outcome over a six-month period

**DOI:** 10.1371/journal.pone.0225303

**Published:** 2019-11-22

**Authors:** Perrine Molmy, Nicolas Vangrunderbeeck, Olivier Nigeon, Malcolm Lemyze, Didier Thevenin, Jihad Mallat

**Affiliations:** 1 Intermediate Care Unit, Centre Hospitalier de Lens, Lens, France; 2 Intensive Care Unit, Centre Hospitalier de Lens, Lens, France; 3 Respiratory & Infectious Diseases Unit, Centre Hospitalier de Lens, Lens, France; 4 Department of Critical Care Medicine, Critical Care Institute, Cleveland Clinic Abu Dhabi, Abu Dhabi, UAE; University of Notre Dame Australia, AUSTRALIA

## Abstract

**Purpose:**

There have been few studies on the limitation of Life Supporting Care (LSC) and Withdrawal of LSC in Intermediate Care Units (IMCUs). We report the prevalence of LSC limited patients in a medico-surgical IMCU over a six-month period, examining the description, outcomes, and patterns of LSC Limitations and Withdrawal of LSC.

**Methods:**

Single center, retrospective observational study in an IMCU of a 500-bed general hospital.

**Results:**

Our study of 404 patients, reported 79 (19.5%, 95%CI: [16.0–23.7]%) being admitted with LSC limitations in the IMCU. This group of LSC limited patients presented with higher chronic and acute severity scores. The most common admission diagnosis of LSC limited patients was acute respiratory failure (51%). Non-invasive ventilation (NIV) was frequently used within this population (39%). Hospital mortality for LSC limited patients was high (53%) and associated with age (OR = 1.07, 95%CI: [1.01–1.13)]), SOFA score (OR 1.29, 95%CI: [1.01–1.64]), and hypoxemic respiratory failure (OR 7.2, 95%CI: [1.27–40.9]). Withdrawal of LSC occurred in 19.5% of cases, often accompanied with terminal sedation with or without NIV removal (43.8%).

**Conclusions:**

Patients with limitation of LSC are frequently admitted into IMCU. Hospital mortality rate was high and associated with age, acute organ failures, and hypoxemic respiratory failure. Life support withdrawal includes palliative sedation with or without NIV discontinuation.

## Introduction

Intermediate Care Units (IMCUs) were implemented in the “90s to provide a graded option between regular ward care and intensive care by providing more frequent monitoring and treatment than is possible on a general ward [1]. The IMCU concept was implemented as a hospital strategy to provide flexibility in patient triage. Though different in their design, one of the objectives of IMCUs is to decompress ICUs, so those resources are appropriately applied to patients who really need them [1, 2]. However, the value of IMCUs has been questioned [3].

The role of IMCUs is far less known than that for ICUs [4]. In our hospital, we believe that IMCUs have an important role in the management of patients who have been placed on limitations of care and withholding or withdrawal of life-supporting care (LSC). However, little is known about the prevalence of such patients in the IMCU settings [5–7]. We, therefore, sought to characterize the prevalence of patients with LSC in our IMCU population, as well as the severity of illness, comorbidities, transitions to the withdrawal of support, and in-hospital mortality.

The first objective of this study was to estimate the prevalence of patients with limitation of LSC treated in the IMCU during the study period, and the factors associated with these limitations. Secondly, we sought to identify the independent risk factors associated with in-hospital mortality in patients with LSC limitation. Third, the causes and modalities of the withdrawal of LSC are also studied.

## Materials and methods

### Ethics statement

This study was approved and authorized by the Lens General Hospital Ethics Committee. This study is purely observational and all data were treated anonymously before analyzing, according to the Ethics’ Committee conditions. Consent to participate was not applicable. No funding was received for this study.

### Study design

Single center retrospective study.

### Source of population

All patients admitted into the IMCU of Lens General Hospital between January 1^st^ and June 30^th^ 2012 were included. The Lens general hospital IMCU is a separate 12-bed unit, distinct from the ICU. The medical team in charge of patients includes Anesthesiologists, and Emergency physicians. The structure of medical staffing is detailed in S1 Table. The nurse: Patient ratio is 1:3. There is no Intensive Care physician dedicated specifically to the IMCU. The intensivists are responsible for determining a patient’s admission or refusal. The Lens general hospital is an acute general hospital with 500 beds that serves a population of 400,000 composed of people living in both rural and urban areas in the North of France. The hospital has an 8-bed Coronary Unit with its own reception. In addition, the Emergency Department possesses a 12-bed unit for short stays to accommodate and monitor the health of less severe patients.

### Data sources

Medical files of all patients admitted into the IMCU were retrieved from the hospital records. Clinical data including medical history and clinical presentation at entry which were extracted from these documents. Laboratory data was recuperated from the hospital electronic database, except in the case of blood gas analysis data which is reported in the daily patient’s care record.

### Definitions and procedures for limitation and withdrawal of life supporting care

**Fig 1:**
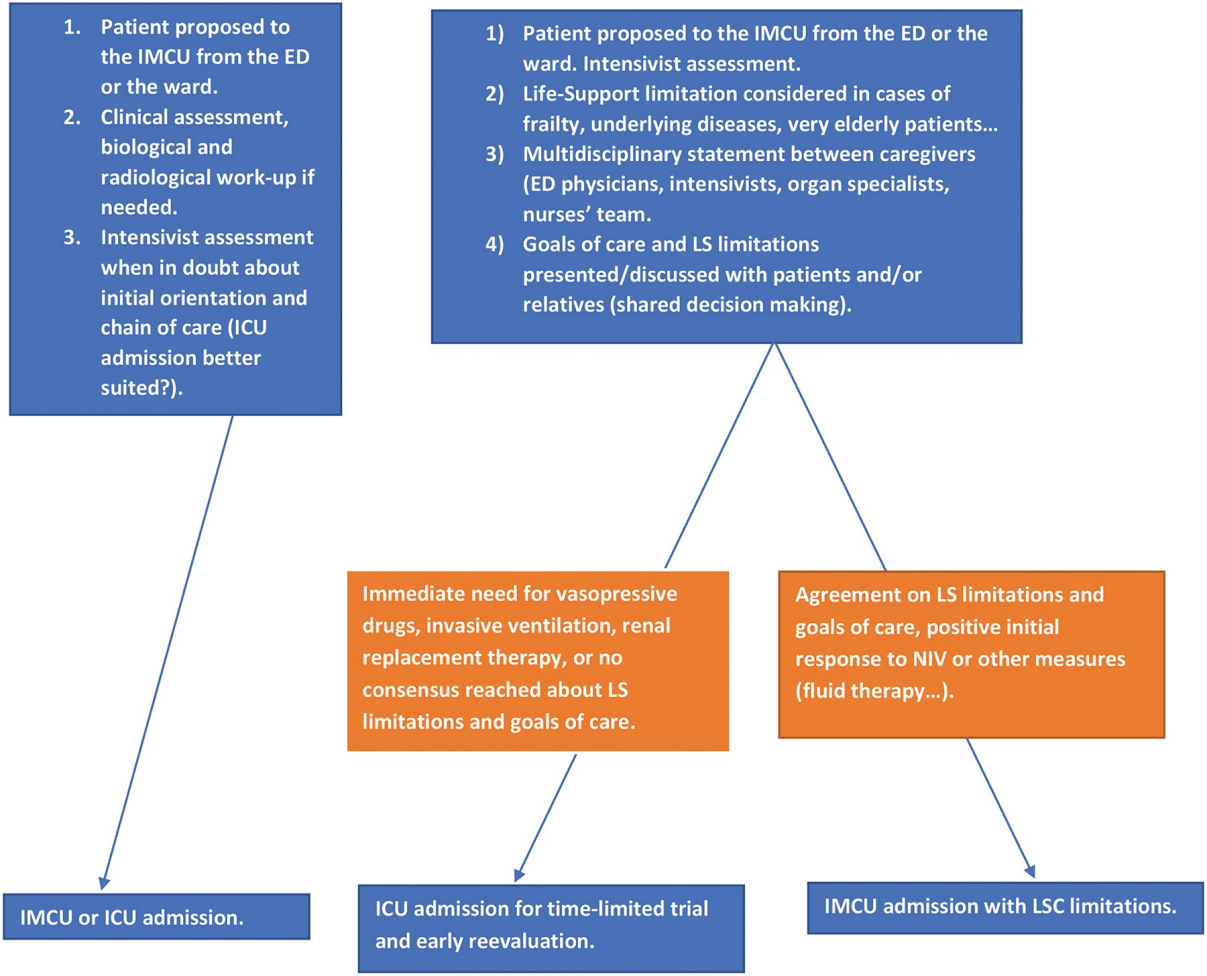
Process of admission (from Emergency Department or Ward), and life-support care limitations if considered. ICU, Intensive Care Unit; IMCU, Intermediate Care Unit.

Limitation of LSC was defined as a decision to not start or increase a life-sustaining treatment. Written "do not Intubate" orders, decisions to not begin vasopressor support, extra-renal replacement therapy or surgery were the conditions of limitation of LSC. Withdrawal of LSC was defined as a decision to actively stop an intervention that was necessary to keep a patient alive through artificial organ supply, including vasopressor support, oxygen-therapy, mechanical/invasive or NIV, and blood transfusions. Limitations of life-supporting care in our hospital includes two components. For patients requiring urgent invasive life-support care at the ED or before hospital admission, the common strategy is an “ICU trial” of few days of intensive care with mandatory reevaluation. Patients admitted directly to the IMCU have limitations of life, and supporting care already anticipated, with limited time to review previous assessment of patients’ characteristics and collegial decisions [8]. For most IMCU patients, these limitations include no use of mechanical ventilation, vasopressors or extra-renal replacement, with NIV being the exception. The process of IMCU admission and limitations of life support are exposed and detailed in Fig 1.

All the limitation and withdrawal of LSC orders were decided through a collegial decision of at least two senior physicians (intensivists, ED physicians, or other specialists or general practitioners) being involved. The decision-making process followed the guidelines of the French Intensive Care Society [9], including patient advance directives when available, even if this is uncommon in France [8]. Other factors involved in the process include multidisciplinary agreement, relatives’ information, and traceability on the medical chart. The document used to follow the LSC limitation or withdrawal process is reported in S1 Text.

Withdrawals of LSC were decided through multidisciplinary agreement, a search of patients’ advance directives or refusal to sustain LSC measures, and relatives ‘information and consent. The decision process includes discussions with attending nurses’ team and family, providing information on the procedures for withdrawal of LSC, including cessation of supportive care and/or sedative treatments according to the French law and guidelines [8]: hospitalization in the IMCU is discussed primarily when an ED or a ward physician proposes a patient to the ICU team for admission. A multidisciplinary consultation is held and includes, when possible, patients’ information and agreement. Goals of care and means are exposed to the nurses’ team to confirm the right course of action. When limitation of life-supporting care is considered, patients and/or their family are informed of the options and possible expectations regarding anticipated outcomes. Before admission, these decisions are written on the patient’s medical file. In the IMCU, the decisions of limitations or withdrawal are compiled in a special section of the medical file (S1 Text).

Limitation and withdrawal of LSC is considered to have commenced once a senior physician transcribed the first notification in the medical file, whether this decision was made in the ED or the ward before admission to the IMCU, or during the IMCU stay.

### Data collection

The following data were recorded from all patients’ files: age, gender, admission category (medical, scheduled or unscheduled surgery), primary symptom or diagnosis at IMCU admission, unit of origin (Emergency Department, ICU, ward or exterior unit). Underlying conditions and autonomy (chronic diseases, malignant tumor, neoplasia with metastasis) were reported and were appreciated through Charlson comorbidity score [10,11] and Knaus index [12,13] (S2 Table). Severity at admission was assessed on the first IMCU day through calculation of Simplified Acute Physiology Score (SAPS) II, Sequential Organ Failure Assessment (SOFA) score. SAPS II score without age was also recorded to estimate the severity without the influence of age. Comorbidities were defined as the existence of more than one chronic organ failure (chronic respiratory or renal insufficiencies, liver cirrhosis, and congestive heart failure) or as the association of a chronic organ failure with neoplastic disease, malnutrition or dementia.

The primary limitation of LSC treatment in the IMCU (NIV, vasopressor support, blood transfusions), the length of stay in the IMCU and the hospital were recorded. Mortality was registered in the IMCU and during the hospital stay.

### Statistical analysis

Data are expressed as mean ± SD when they are normally distributed, or as median [25–75%, interquartile range, (IQR)] when they are non-normally distributed. The normality of data distribution was assessed using the Kolmogorov-Smirnov test, and visually by histogram. Comparisons of values between different groups of patients were performed by two-tailed Student’s t test, or Mann–Whitney U-test, as appropriate. Analysis of categorical data was performed using the χ^2^ and Fisher’s exact tests. Multivariable logistic regression (entry p<0.25) [14] was used to identify significant independent predictors that were associated with the limitations of LSC, and with the hospital mortality in the LSC limited patients group. Goodness-of-fit of the model was assessed using the Hosmer–Lemeshow test. The potential problem of co-linearity was evaluated before running the analysis. Statistical analysis was performed using SPSS for Windows release 17.0 software (Chicago, Illinois, USA). p<0.05 was considered statistically significant. All reported P values are 2-sided.

## Results

### Study population

A total of 404 consecutive admitted patients were studied. Global population patients’ characteristics are presented in Table 1. Limitation of LSC was registered for 19.5% (95%CI: [16.0–23.7]%) of patients; it was determined before or at IMCU admission for 70% of these patients, and during the stay for 30%. Withdrawal of LSC was subsequently decided for 20% of patients with LSC limitation.

**Table 1:** General characteristics of IMCU patients (n = 404).

**Age [median (IQR); year]**	**64 [53–76]**
**Sex Male, n (%)**	**266 (65.8)**
**Admissions diagnoses**	
**Medical diagnosis, n (%)**	**248 (61.4)**
**Acute respiratory failure, n (%)**	**101 (25.0)**
**Digestive tract bleeding, n (%)**	**51 (12.6)**
**Other causes, n (%)**	**96 (23.8)**
**Scheduled surgery admission, n (%)**	**78 (19.3)**
**Unscheduled/urgent surgery, n (%)**	**67 (16.6)**
**Obstetric causes, n (%)**	**11 (2.7)**
**Origin of patients**	
**ICU, n (%)**	**69 (17.1)**
**Emergency Department, n (%)**	**144 (35.6)**
**Ward or exterior units, n (%)**	**191 (47.3)**
**Life supporting care limitations, n (%)**	**79 (19.5)**
**LSC-L in the emergency department or decided at admission in IMCU, n (%)**	**55 (13.6)**
**LSC-L decided during IMCU stay, n (%)**	**24 (6.0)**
**LSC withdrawal, n (%)**	**16 (4)**
**SAPS II [median (IQR]**	**24 [15–32]**
**SAPS II without age [median (IQR]**	**12 [8–19]**
**SOFA score [median (IQR]**	**2 [1–4]**
**Charlson score [median (IQR]**	**5 [2–6]**
**Knaus index [median (IQR]**	**3 [2–3]**
**IMCU LOS [median (IQR); day]**	**4 [2–6]**
**Hospital LOS [median, (IQR); day]**	**12 [8–22]**
**IMCU mortality, n (%)**	**30 (7.4)**
**In-hospital mortality, n (%)**	**62 (15.3)**

**ICU**, Intensive Care Unit; **IMCU**, Intermediate Care Unit; **LSC**, Life Supporting Care; **LSC-L**, Life Supporting Care Limitation; **SAPS II**, Simplified Acute Physiology Score; **SOFA**, Sequential Organ failure Assessment; **LOS**, Length of stay.

### Characteristics of patients

The comparisons between LSC limited and LSC unlimited patients are presented in Table 2. Patients with Limitations of LSC were globally older, presented with more severe chronic illness and higher severity at admission than LSC unlimited patients. The Knaus index was also higher in LSC limited patients compared to patients without LSC limitations (4 [3–4] vs. 3 [2–3], p<0.001; respectively), reflecting greater weakness and dependence. The Admission category was mostly medical in LSC limited patients, with acute respiratory failure as the principal diagnosis. NIV was the most frequent LSC treatment. Other LSC invasive supports were anecdotical (data not shown).

**Table 2:** Comparison of patients with or without LSC limitation: Univariate analysis.

	Limited LSC (patient	Unlimited LSC patient	*p*
**n**	**79**	**325**	
**Age [median (IQR); year]**	**73 [62–82]**	**62 [51–74]**	**<0.001**
**Sex male, n (%)**	**52 (66)**	**214(65.8)**	**0.98**
**Severity and comorbidity scores**			
**SAPS II [median (IQR)]**	**35 [27–40]**	**21 [13–28]**	**<0.001**
**SAPS II without age [median (IQR)]**	**21 [13–26]**	**10 [6–16]**	**<0.001**
**SOFA score [median (IQR)]**	**4 [2–6]**	**2 [2–3]**	**<0.001**
**Charlson [median (IQR)]**	**7 [5–8]**	**4 [2–6]**	**<0.001**
**Knaus index [median (IQR)]**	**4 [3–4]**	**3 [2–3]**	**<0.001**
**Admission diagnoses, n (%)**			
**Medical diagnosis**	**71 (90.0)**	**177 (54.5)**	**<0.001**
**Hypoxemic Respiratory Failure**	**16 (20.3)**	**43 (13)**	**0.19**
**Hypercapnic Respiratory failure**	**24 (30.4)**	**18 (5.5)**	**<0.001**
**Digestive tract bleeding**	**11 (14)**	**40 (12.3)**	**0.75**
**Other causes**	**20 (25.3)**	**76 (23.4)**	**0.55**
**Scheduled surgery admission**	**4 (5)**	**74 (23)**	**<0.001**
**Unscheduled/urgent surgery**	**4 (5)**	**63 (19.4)**	**0.002**
**Obstetric causes**	**0 (0)**	**11 (3.4)**	
**Mortality and length of stay**			
**IMCU LOS [median (IQR); day]**	**5 (3–10)**	**3 (2–5)**	**<0.001**
**IMCU mortality, n (%)**	**25 (32)**	**2 (0.6)**	**<0.001**
**In-hospital mortality, n (%)**	**42 (53)**	**22 (6.7)**	**<0.001**

**ICU**, Intensive Care Unit; **LSC**, Life Supporting Care; **LSC-L**, Life Supporting Care Limitation; **SAPS II**, Simplified Acute Physiology Score; SOFA, Sequential Organ failure Assessment; **LOS**, Length of stay; IMCU, Intermediate Care Unit.

In-hospital mortality was significantly higher in the limited LSC patients compared to patients without LSC limitations (53% vs. 6.7%, p<0.001). Also, the median length of stay in IMCU and the IMCU mortality were both significantly higher in the limited LSC patients’ group (Table 2).

### Factors associated with limitation of LSC orders

In the univariate analysis, 11 variables were associated with LSC limitation with p<0.25 for entry into multivariate models (Table 2). Multivariable logistic regression analysis with LSC limitation as the dependent variable was then performed. For reason of co-linearity between SAPS II score and SAPS II without age score, and because medical diagnosis and acute respiratory failure types were not independent, nine variables were finally included in the model (age, SAPS II without age score, SOFA score, Charlson score, Knaus score, hypoxemic and hypercapnic acute respiratory failures, scheduled post-surgery admission, and urgent post-surgery admission). Among these variables, high SOFA score, a high degree of comorbidities (Charlson score) and low autonomy were significantly associated with LSC limitation (Table 3). Surgical patients admitted into IMCU were less likely to have LSC limitation, whereas hypercapnic acute respiratory failure type was strongly associated with limitation of LSC. Limitation of LSC was decided before or early after IMCU admission. The median delay of LSC limitations decisions was less than 1 day (0–1). Alleged reasons for LSC limitations decisions were mainly advanced age (4.5%), limited autonomy (50.6%), comorbidities (88.6%), and poor outcome of underlying diagnosis (78.5%). Several of these causes were found in most cases (88.6%).

**Table 3:** Comparison of patients with or without LSC limitation: Multivariable analysis.

	OR (95% CI)	*p*
**Age**	**1.00 (0.97–1.04)**	**0.93**
**Severity scores**		
**SAPS II without age**	**1.00 (0.97–1.05)**	**0.75**
**SOFA score**	**1.34 (1.10–1.63)**	**0.003**
**Charlson score**	**1.42 (1.17–1.72)**	**0.001**
**Knaus index**	**12.40 (5.64–27.34)**	**<0.001**
**Admission diagnoses**		
**Hypoxemic acute respiratory failure**	**0.99 (0.36–2.72)**	**0.98**
**Hypercapnic acute respiratory failure**	**4.40 (1.62–12.00)**	**0.001**
**Post- surgical admission**	**0.37 (0.09–1.51)**	**0.16**
**Unscheduled/urgent surgery**	**0.22 (0.048–0.98)**	**0.048**

**SAPS II**, Simplified Acute Physiology Score; **SOFA**, Sequential Organ failure Assessment **CI**, confidence interval

### Factors associated with hospital mortality in LSC limited patients

The hospital mortality rate of LSC limited patients was 53% (95%CI: [42–64]%) (Table 4). Patients with LSC limitations who died during the hospital stay were older, had higher SAPS II and SOFA scores, and suffered from much more acute hypoxemic respiratory failure compared with LSC limited patients who survived (Table 4). IMCU patients with digestive tract bleeding and LSC limitations had a good survival rate. Age, SOFA score, and acute hypoxemic respiratory failure cause were found to be independent predictor factors of hospital mortality in LSC limited patients (Table 5).

**Table 4:** Survival of patients with LSC limitation: Univariate analysis.

	Deceased during hospital stay	Surviving hospital stay	*p*
**n**	**42**	**37**	
**Age [median (IQR); year]**	**76 [64.4–84.0]**	**64 [59–79]**	**0.006**
**Sex male, n(%)**	**27/42 (64.3)**	**25/37 (67.6)**	0.76
**Severity score**			
**SAPS II [median (IQR)]**	**37.5 [34–43]**	**30 [24–35]**	**<0.001**
**SAPS II without age [median (IQR)]**	**21.5 [16.5–29]**	**18 [12–23]**	**0.02**
**SOFA score [median (IQR)]**	**4 [3–7]**	**3 [2–5]**	**0.019**
**Charlson score [median (IQR)]**	**7 [5–8]**	**7 [5–8]**	0.75
**Knaus index [median (IQR)]**	**4 [3–4]**	**4 [3–4]**	0.89
**Admission diagnoses n (%)**			
**Medical diagnosis, n (%)**	**39/42 (93)**	**32/37 (86.5)**	0.35
**Hypoxemic acute respiratory failure, n(%)**	**14/42 (33.3)**	**1/37 (2.7)**	**<0.001**
**Hypercapnic acute respiratory failure, n(%)**	**11/42 (26.2)**	**13/37 (35.1)**	0.39
**Digestive tract bleeding, n(%)**	**2/42 (4.8)**	**9/37 (24.3)**	**0.02**
**Other causes, n (%)**	**12/42 (28.6)**	**9/37 (24.3)**	0.67
**Scheduled surgical admission, n(%)**	**0/42 (0)**	**4/37 (10.8)**	**0.04**
**Unscheduled/ urgent surgery, n(%)**	**3/42 (7.1)**	**1/37 (2.7)**	0.62

**SAPS II**, Simplified Acute Physiology Score; **SOFA**: Sequential Organ Failure Assessment.

**Table 5:** Mortality of patients with LSC limitation: Multivariable analysis.

Factors associated with survival	OR (95% confidence interval)	*p*
**Age**	**1.06 (1.01–1.12)** **(0.99–1.10)**	**0.018**
**Severity scores**		
**SAPS II without age**	**1.06 (0.99–1.14)**	0.08
**SOFA score**	**1.29 (1.01–1.66)**	**0.046**
**Admission diagnoses**		
**Hypoxemic acute respiratory failure**	**17.8 (1.76–180.2)**	**0.01**
**Digestive tract bleeding**	**0.39 (0.64–2.37)**	0.31

**SAPS II**, Simplified Acute Physiology Score; **SOFA**, Sequential Organ Failure Assessment

### Comparisons between LSC limitations before and after IMCU admission in LSC limited patients

Amongst LSC limited patients, 55 (70%) had LSC limitations before IMCU admission compared to 24 (30%) patients who had LSC limitations after their admissions into IMCU Despite the small sample size, we performed an analysis that showed no significant differences between the two sub-groups regarding age, sex, severity scores, and in-hospital mortality rate. Medical diagnoses, particularly hypercapnic failure, were more frequently associated with LSC limitations before than after IMCU admission (S3 Table).

### Characteristics of the 15 patients died after with withdrawal of LSC and modalities of withdrawal of LSC

Description of patients’ patterns is reported in S4 Table. In summary, median age was 75.5 years [61.8–81.5], Charlson score of 6.5 [5–7], Knaus index of 4 [3–4], and SAPS II of 38 [27–40]. Most of these patients were admitted for medical diagnosis, and presented with several comorbidities. No comparison with the LSC limited patients without withdrawal of LSC was made due to the small sample size. Cessation of LSC was realized soon after the admission (median delay 1 day, IQR 0–3) for most patients and included terminal sedation (88% of cases), with or without discontinuation of NIV (44% of NIV discontinuation cases).

## Discussion

The study’s major findings were: 1) limitation of LSC was common in the IMCU and was often decided before patients’ admission. It was associated with a poor general condition or limited autonomy, comorbidities, however not with age. LSC limited patients presented with higher acute severity than unlimited LSC patients; 2) hospital mortality of LSC limited patients was high (42 patients, 53%) and related to age, preexisting organ failures or severe diseases, and acute severity. In LSC limited patients, hypoxemic respiratory failure cause was associated with high mortality, but not the hypercapnic respiratory failure cause. Finally, 20% of LSC limited patients (all of them under NIV) received terminal sedation as the main mode of LSC withdrawal. Half of these patients were weaned from NIV after LSC withdrawal decision.

Our results outline the daily hospital cycle of LSC limited patients’ admission to the IMCU. Few data are available to date on this topic [5–7], although, IMCU currently represents a significant part of acute care resources in Europe and Northern America [2, 15, 16–19].

In our study, LSC limitation decisions appear to be more associated with patterns of frailty than with age alone, as in previous studies [17–22]. Another interesting observation is that LSC limitation was found to be more frequently related to “medical admission patients” than to surgical admission causes. Our results are in accordance with what was reported earlier but in ICU [19, 20]. Strikingly, the LSC-limited patients also presented with higher severity scores in comparison to unlimited LSC patients admitted into the IMCU. IMCUs are not designed to receive and treat patients with multiple organ failures and high severity but appear to do so when a decision of LSC limitation is made [18].

Half of the LSC limited patients died during hospitalization. Accordingly, it is important to establish conditions that are associated with mortality to estimate when IMCU admission might likely be beneficial [23]. Independent predictor factors for hospital mortality in LSC limited patients were older age, chronic and acute severity, and admission patterns, with a clear gap between prognosis of hypercapnic and hypoxemic acute respiratory failures.

This last observation may reflect the difference in success probability of NIV according to the type of respiratory failure, as demonstrated previously [23–27], since NIV was the main organ support used in these patients. Thus, the use of NIV, rather than an “NIV for DNI patient with respiratory failure” approach, should imply thoughts about etiologic diagnoses, goals of care, and patients’ response [28, 29]. This point is important, since NIV-limited use in LSC limited patients with a poor predicted outcome might be considered as “unreasonable” if it doesn’t support patient’s relief, or necessitates more constraints [16].

The impact of chronic organ failures in IMCU patients underscores the importance of preexisting pathologic conditions in determining the objectives of LSC care, a fact that is often underestimated in the ICU setting [30–32].

Moreover, the poor prognosis of the majority of severe patients admitted to the IMCU with LSC limitations raises questions about the aims of care for them. Is it reasonable to hope for recovery for the most frail and severe patients? [22, 30, 31]. The impact of bed occupancy by such patients has been studied in the ICU, and it has been suggested that it could harm other patients through unavailability of ICU care [32]. It is also worthwhile to note that these “futile” admissions for end-of-life care in ICUs generate significant costs [33–35]. These problems have to be looked at in the IMCUs because of their role in the chain of care for critically ill patients [4, 13, 36].

Another key observation is that the care of such patients with unlikely hope for recovery is the potential perception of “inappropriate care” by medical and nursing teams [37, 38], which has been linked to staff burn-out and retention [37, 39].

Other factors that could be linked to associated staff grievances and personal struggles are the patterns of LSC withdrawal. NIV was discontinued in nearly a half of patients (7 patients, 44%) with LSC withdrawal, a fact that could reflect its use as a palliative measure to relieve dyspnea [25, 29], or physicians’ differences in modalities of stopping ventilation [40, 41].

Our study has several limitations. First, it was a retrospective monocentric, time-limited experience, and thus, our data could not be generalized without previously extended research. Nevertheless, although data were obtained in 2012, the scenario that is addressed has not changed in our institution and is not outdated. Secondly, the proper weight of comorbidities in IMCU patients’ outcome remains also difficult to establish, since all these patients presented with high Charlson scores, emphasizing their frailty. Some organ failures may be more involved in hospital mortality, but this has still to be established. Thirdly, LSC limitations in the other units at the same time were not recorded. Accordingly, it is hard to appreciate which part of LSC limitation and end of life process the IMCU is providing in the global acute care setting. Long-term survival or quality of life after hospital stay could not be studied either. Fourthly, use of NIV as a palliative measure [25] was not recorded, because the medical files did not inform in which goal of care NIV was performed. Finally, the small sample size may have overlooked factors associated with LSC limitation or mortality.

### Conclusions

We observed a 19.5% of patients with limitation of LSC admitted into our IMCU. Patients with heavy medical conditions and high severity generally have a poor prognosis. Nevertheless, some patients, notably those with hypercapnic failure, benefit from IMCU admission, with NIV as a key therapy. Poor outcomes in the most critical patients, with several comorbidities and acute organ failures, raises questions about the appropriateness of care and subsequent impact on staff.

## Supporting information

S1 Table (DOCX)

S2 Table (DOCX)

S3 Table (DOCX)

S4 Table (DOCX)

S1 Text (DOCX)

S1 Data (XLSX)

## Abbreviations

ED: Emergency department
ICU: Intensive Care Unit
IMCU: Intermediate Care Unit
LOS: Length of stay
LSC: Life Supporting Care
LSC-L: Life Supporting Care Limitation
NIV: Non-invasive Ventilation
SAPS II: Simplified Acute Physiology Score
SOFA: Sequential Organ failure Assessment
