# Supplementary material for: Patients with limitation or withdrawal of life supporting care admitted in a medico-surgical intermediate care unit: Prevalence, description and outcome over a six-month period
Source: PLoS One. 2019 Nov 22;14(11):e0225303. doi: 10.1371/journal.pone.0225303 (PMC6874297; doi:10.1371/journal.pone.0225303)
Supplement: S1 Table — (DOCX) [file pone.0225303.s001.docx]

**S1 Table: IMCU structure and process for Admission and Life-Support Limitation**

**Structure of medical staffing**:

| **Intensivists:**  In charge of admission triage, administrative responsibility, weekly meetings for medical guidance and to establish goals of care, technical help in emergencies, night coverage for « medical » patients | **ED physicians:** in charge of daily care of « medical » patients (6 beds), discharge letters, family meetings |
| --- | --- |
|  | **Anesthesiologists:** in charge of « surgical » patients (6 beds for post-operative care or deteriorating surgical wards patients), for daily care and night coverage, family meetings, discharge letters |
