# Supplementary material for: Patients with limitation or withdrawal of life supporting care admitted in a medico-surgical intermediate care unit: Prevalence, description and outcome over a six-month period
Source: PLoS One. 2019 Nov 22;14(11):e0225303. doi: 10.1371/journal.pone.0225303 (PMC6874297; doi:10.1371/journal.pone.0225303)
Supplement: S2 Table — (DOCX) [file pone.0225303.s002.docx]

**S3 Table: Knaus Index calculation**

| Class A | Normal health status |
| --- | --- |
| Class B | Moderate activity limitation |
| Class C | Severe activity limitation due to chronic disease |
| Class D | Bedridden patient |
