# Supplementary material for: Patients with limitation or withdrawal of life supporting care admitted in a medico-surgical intermediate care unit: Prevalence, description and outcome over a six-month period
Source: PLoS One. 2019 Nov 22;14(11):e0225303. doi: 10.1371/journal.pone.0225303 (PMC6874297; doi:10.1371/journal.pone.0225303)
Supplement: S3 Table — (DOCX) [file pone.0225303.s003.docx]

**S4 Table**

**Comparisons between LSC limitations before and after IMCU admission, N=79**

|  | LSC limitations before IMCU admission | LSC limitations after IMCU admission | *p* |
| --- | --- | --- | --- |
| n | **55** | **24** |  |
| Age, [median (IQR); year] | **73.0 [62 – 81.0]** | **73.5 [61.2 -84.2]** | **0.74** |
| Sex male, n (%) | **35 (63.6)** | **17 (70.8)** | **0.53** |
| Severity score |  |  |  |
| SAPS II, [median (IQR)] | **35.0 [28.0 – 42.0]** | **34.5 [21.7 – 38.5]** | **0.31** |
| SAPS II, without age [median (IQR)] | **21.0 [15.0 – 26.0]** | **18.0 [8.5- 26.0]** | **0.16** |
| SOFA score, [median (IQR)] | **4.0 [2.0 – 6.0]** | **4.0 [2.0 – 6.0]** | **0.99** |
| Charlson score, [median (IQR)] | **7.0 [5.0 – 8.0]** | **7.0 [5.0 – 8.0]** | **0.95** |
| Knaus index, [median (IQR)] | **4.0 [3.0 – 4.0]** | **3.5 [3.0 – 4.0]** | **0.27** |
| In-hospital mortality, n (%) | **28 (50.9)** | **14 (58.3)** | **0.53** |
| Admission diagnoses, n (%) |  |  |  |
| Medical diagnosis, n (%) | **52 (94.5)** | **19 (79.2)** | **0.037** |
| Hypoxemic acute respiratory failure, n (%) | **12 (21.8)** | **3 (12.5)** | **0.53** |
| Hypercapnic acute respiratory failure, n (%) | **23 (41.8)** | **1 (4.2)** | **<0.001** |
| Digestive tract bleeding, n (%) | **7 (12.7)** | **4 (16.7)** | **0.73** |
| Other causes, n (%) | **10 (18.2)** | **11 (45.8)** | **0.014** |
| Scheduled surgical admission, n (%) | **1 (1.8)** | **3 (12.5)** | **0.081** |
| Unscheduled/ urgent surgery, n (%) | **2 (3.6)** | **2 (8.3)** | **0.58** |

**SAPS II**, Simplified Acute Physiology Score; **SOFA**, Sequential Organ Failure Assessment; **LSC**, Life Supporting Care; **IMCU**, Intermediate Care Unit.
