# Supplementary material for: Patients with limitation or withdrawal of life supporting care admitted in a medico-surgical intermediate care unit: Prevalence, description and outcome over a six-month period
Source: PLoS One. 2019 Nov 22;14(11):e0225303. doi: 10.1371/journal.pone.0225303 (PMC6874297; doi:10.1371/journal.pone.0225303)
Supplement: S4 Table — (DOCX) [file pone.0225303.s004.docx]

**S5 Table. Characteristics of patients with LSC withdrawal, Withdrawal modalities, (n= 15)**

| Age [median (IQR); year] | 75.5 (61.8-81.5) |
| --- | --- |
| Sex (Male / Female) n (%) | 10 (62.5) |
| **Severity and Comorbidity scores** |  |
| SAPS2 [median (IQR)] | 38 (33.8 41.3) |
| SAPS2 without age [median (IQR)] | 21 (17.3- 27.5) |
| SOFA [median (IQR)] | 5 (3- 7) |
| Charlson [median (IQR)] | 6.5 (5-7) |
| Knaus Index A=1 B=2 C=3 D=4 [median (IQR)] | 3 (3-4) |
| Medical diagnosis at admission n (%)  Hypoxemic acute respiratory failure n (%)  Hypercapnic acute respiratory failure n (%)  NIV n (%) | 11 (68.8)  5 (31.3)  7 (43.8)  9 (56.3) |
| Time between admission and decision of LSC withdrawal | 1 (1-2.3) |
| **Reasons alleged for Withdrawal n (%)** |  |
| Patient’s refusal to sustain LSC therapies | 3 (18.8) |
| Unefficiency of LSC therapies | 16 (100) |
| **Evolution** |  |
| NIV discontinuation n (%) | 7 (43.8) |
| Palliative sedation n (%) | 14 (87.5) |
| IdtCU length of stay [median (IQR); day] | 5.5 (3.8-7) |
| Hospital length of stay [median (IQR); day] | 9 (4.8-13.3) |
| Decease in the IdtCU n (%) | 16 (100) |

SAPS II: Simplified Acute Physiology Score. SOFA: Sequential Organ failure Assessment. SAPS II without age: Simplified Acute Physiology Score, with retrieved points accorded to the age of patients.

*Medical diagnoses: any diagnosis at admission different from post-surgery admission, trauma or post-partum management.
