# Supplementary material for: Patients with limitation or withdrawal of life supporting care admitted in a medico-surgical intermediate care unit: Prevalence, description and outcome over a six-month period
Source: PLoS One. 2019 Nov 22;14(11):e0225303. doi: 10.1371/journal.pone.0225303 (PMC6874297; doi:10.1371/journal.pone.0225303)
Supplement: S1 Text — (DOCX) [file pone.0225303.s005.docx]

**S2: Limitations or Withdrawal of LSC IMCU document**

**Limitation or Withdrawal of life-support treatments**

**INTERMEDIATE CARE UNIT**

**Article L 1110-5 of the Public Health Code** “Any person…[]… has the right to receive the most suitable care”
**Law 2005-370 of April the 22^nd^ 2005 relating to the right of the patients and the end-life time** “These acts should not be continued by an unreasonable obstinacy. When they appear useless, disproportionate or having for only effect the artificial maintenance of life, they can be suspended or not be undertaken. In this case, the doctor saves the dignity of a dying patient and ensures the quality of his life by providing cares described in article 1110-10”
**Article L1110-10 of the public Health Code** “Palliative care is active and continuous care…[]. They aim to relieve pain, to alleviate psychic suffering, to save dignity of the sick person and to support her entourage.

**Patient Name**: **First name:** **Birth date:**

**Medical history:**

**Former autonomy:
□** Complete Autonomy □ Relative autonomy (need for assistance) □ Dependant □ Invalid

**Reason of hospitalization:**

**Medical summary:**

**Therapeutic project:**

**Motivations of this meeting:
□** medical request **□** caregivers request **□** patient request **□** family or close relations request

**Date of the meeting:**

**People present:**

**Advisory opinion: □** no **□** yes : Dr ……………………………Specialty: ………………………..

**Limitation of life-sustaining treatments:**

**□** No cardiopulmonary resuscitation **□** No introduction of vasopressor agents **□** No admission in ICU for hemodialysis or mechanical ventilation **□** No surgical recovery **□** No antibiotherapy **□** No NIV **□** No massive transfusion

**Life-sustaining treatments withdrawal:**

**□** NIV **□** Fluid replacement **□** Vasopressor agents **□** Antibiotherapy **□** Enteral or parenteral nutritional support

**Patient comfort care:**

**□** Sedation (benzodiazepines) **□** Analgesia (morphine**)
□** Bronchial secretions reduction (scopolamine) **□** Others
 **□** Blood tests and imaging withdrawal **□** Ablation of invasive devices  **□** naso-enteral tube **□** vesical tube **□** psychological support **□** request for mobile palliative team intervention

**Arguments justifying these decisions:**

**□** Patient anticipated directives **□** Initial short term poor prognosis **□** Age **□** Comorbidities **□** Faded former autonomy **□** Very faded future autonomy **□** Therapeutic inefficiency **□** Hopeless therapeutic**s
□** Useless aggressive treatment felt by medical staff or caregivers **□** Refusal care by patient **□** Patient suffering **□** Useless aggressive treatment felt by family or close relations

**Patient previously consultated: □** Yes **□** No

Expressed Will: ………………………………………………………………………………………………………………………..

**Family previously consulted:** **□** Yes **□** No

Name, first names, relationship: ……………………………………………………………………………………

……………………………………………………………………………………

……………………………………………………………………………………

Expressed Will: ………………………………………………………………………………………………………………………..

**Present staff names and signatures**

Clear explanations and precise datas given to the patient (if capable) and/or to the family or close relations

By: ………………………………………………… Date: ……………………………………………..
